# Sarcopenia predicts immune-related adverse events due to anti-PD-1/PD-L1 therapy in patients with advanced lung cancer

**DOI:** 10.3389/fonc.2024.1450020

**Published:** 2024-09-23

**Authors:** Dinglong Xue, Ning Li, Jiaxin Yang, Kaiya Men, Lijun Li, Hao Jiang, Xu Zhao, Shuai Zhang

**Affiliations:** Department of Medical Oncology, Harbin Medical University Cancer Hospital, Harbin, China

**Keywords:** immune checkpoint inhibitor, immune-related adverse events, sarcopenia, non-small cell lung cancer, L3-SMI

## Abstract

**Introduction:**

Immune checkpoint inhibitors (ICIs) have revolutionized the treatment of a number of patients with advanced cancer, and while this has resulted in increased survival times, it has also led to the emergence of novel immune-related adverse events (irAEs). In individuals with advanced cancer, sarcopenia is a significant symptom of cachexia and is linked to poor nutritional status and increased mortality. The present study aimed to evaluate sarcopenia and other risk variables that can affect the emergence of irAEs in patients with lung cancer.

**Methods:**

A single-center retrospective analysis of 129 patients with advanced lung cancer treated with programmed cell death protein-1 (PD-1)/programmed cell death ligand-1 (PD-L1) checkpoint inhibitors was conducted from August 2020 to August 2022. Data on baseline characteristics and adverse events of participants were collected. Computed tomography was used to determine the skeletal muscle index at the third lumbar vertebra (L3-SMI) and whether sarcopenia is present.

**Results:**

The median age of all participants was 60 years old (range, 52-66 years), with men accounting for 68.9% of the total patient cohort. The present study showed that 44 (34%) participants presented with any degree of irAEs, and 79 (61.2%) patients presented with sarcopenia. There were no statistically significant differences in baseline characteristics, such as age and sex, between patients who presented with irAEs and those without irAEs. Using logistic regression analysis, individuals with sarcopenia were 2.635-times more likely to experience any grade of irAEs than those without sarcopenia.

**Discussion:**

irAEs are prevalent side effects of PD-1/PD-L1 inhibitor therapy for patients with cancer. By diagnosing and treating sarcopenia early, it is possible to lower the potential risk of irAEs in patients with advanced cancer. Furthermore, sarcopenia can be utilized as a predictor of irAEs.

## Introduction

The development of immune checkpoint inhibitors (ICIs) has fundamentally altered the way advanced malignancies are treated and has given patients more therapy options. Therefore, it is considered a major development in the field of cancer treatment. ICIs have been shown to be effective in treating cancers and significantly prolong survival (1–6). Nivolumab, pembrolizumab, atezolizumab, durvalumab monotherapy or combination therapy have become the standard first-line treatments for non-small cell lung cancer (NSCLC) (7). ICIs block inhibitory signals from the immune system by binding to inhibitory receptors expressed on T cells or their ligands, reversing immune escape, promoting tumor cell death and exerting anti-tumor effects (8). The unique anti-tumor mechanisms of ICIs carry a risk of overactivating the immune system, leading to specific and extensive toxicity, which are considered immune-related adverse events (irAEs). A MATE analysis showed a 26.82% incidence of irAEs and 6.10% incidence of severe irAEs in all patients receiving anti-programmed cell death protein-1 (PD-1)/programmed cell death ligand-1 (PD-L1) therapy. Reportedly, irAEs mainly affect the skin, endocrine system, digestive system, respiratory system and urinary system (9), and mortality rates associated with irAEs range from 0.36-1.23% (10). Therefore, clinicians need to identify risk factors for the development of irAEs and intervene as early as possible to improve patient prognosis.

Previous studies have shown that obesity, a high body mass index (BMI), the presence of autoimmune disease at diagnosis, low levels of neutrophils, lower performance status score and cancer type are all associated with the development of irAEs (11–14). Sarcopenia, as a specific manifestation of decreased physical status, is defined as a loss of muscle mass and function (15) and constitutes an important component of cancer cachexia. Sarcopenia has been found to be associated with poor clinical outcomes in a variety of diseases and reduce the treatment response to ICIs (15–20). Furthermore, it has been demonstrated that pre-treatment sarcopenia in patients with cancer is independently associated with chemotherapy-induced toxicity. Regarding immunotherapy, patients with sarcopenia and low muscle attenuation (MA) are more likely to experience severe treatment related toxicity with ipilimumab treatment (21). For anti-PD-1 treatment, sarcopenia and high body mass index association has been identified as a risk (22). However, it remains unclear which patients will develop irAEs and little is known about how sarcopenia affects the occurrence of irAEs in patients with advanced cancer.

The present study retrospectively analyzed the data of 129 patients with advanced lung cancer treated with anti-PD-1/PD-L1. The purpose was to investigate whether sarcopenia, as well as other factors, could predict the development of irAEs in patients with advanced lung cancer.

## Materials and methods

### Study design and participants

The present retrospective study involved 129 patients with stage IV NSCLC treated with single-agent anti-PD-1/PD-L1 as a first- or subsequent-line treatment at Harbin Medical University Cancer Hospital (Harbin, China) from August 2020 to August 2022. The exclusion criteria of the patients were as follows: i) Patients who have previously received ICI treatment; ii) have multiple malignancies; iii) have not had abdominal cross-sectional imaging [computed tomography (CT) or PET/CT] within three months of starting treatment; iv) have other autoimmune diseases; v) are receiving other immunosuppressive therapies; vi) have an expected survival of <3 months; and vii) are receiving end-of-life care.

### Data collection

Prior to starting anti-PD-1/PD-L1 therapy, the following participant data were collected: Height, weight, age, sex, the Eastern Cooperative Oncology Group (ECOG) performance status (PS), smoking status, PD-L1 expression, lung cancer histology and anti-PD-1/PD-L1 treatment regimen. BMI was calculated as follows: BMI = weight/height^2^, with height measured in meters and weight measured in kg. The ECOG five-point activity status scale was used to calculate the performance status score. The nutritional status of the participants was classified using the Patient-Generated Subjective Global Assessment (PG-SGA), which is a nutritional status assessment method specially designed for cancer patients and is an effective tumor patient-specific nutritional assessment tool. Through the above evaluation, the total score is calculated, and the patients can be clinically classified into three categories: well-nutritional status (0-3 points), moderate or suspected malnutrition (4-8 points), and severe malnutrition (≥9 points). Retrospective evaluation of irAEs was carried out using Participants’ electronic medical records. In addition, Common Terminology Criteria for Adverse Events (CTCAE; version 5.0) was used for the assessment and classification of irAEs. Depending on the organ/system in which the irAEs occurred, the irAEs were categorized as follows i) Endocrine irAEs (with symptoms such as thyroid disorders and pituitary disorders); ii) hepatic irAEs; iii) pulmonary irAEs; iv) gastrointestinal irAEs (with symptoms such as diarrhea and colitis); v) skin irAEs (with symptoms such as rashes); and vi) other irAEs (such as renal, hematologic or neuromuscular irAEs).

### Determination of sarcopenia

**Figure 1:**
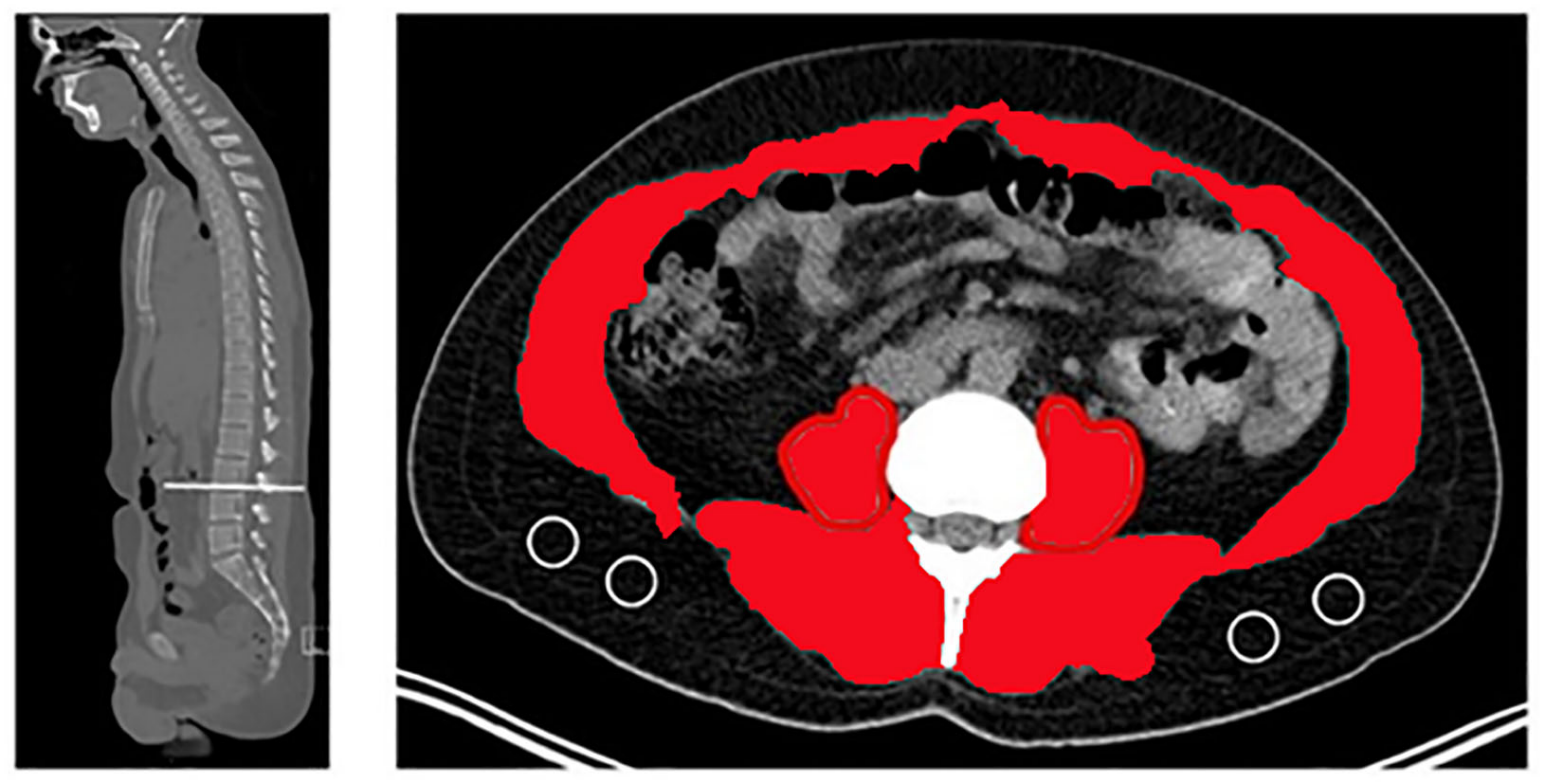
Cross-sectional computed tomographic images at the L3 level.

Computed tomography (CT) scans are the most common disease assessment tool for patients with lung cancer, thus the obtained CT scan data was used to identify sarcopenia. The study sample was limited to CT scan data taken within 3 months before and after starting treatment. CARESTREAM.RIS.GRC (Carestream Health, Inc.) software was used to calculate the cross-sectional area of the psoas muscle at the caudal end of the L3 segment. The cross-sectional area of the L3 skeletal muscle was determined and measured using Hounsfield unit thresholds (29 to +150) (23). The following equation was used to calculate the L3-SMI: L3-SMI (cm^2^/m^2^) = sum of cross-sectional area of skeletal muscle on both sides (cm^2^)/height^2^ (m^2^) (**Figure 1**).

In a previous study, new diagnostic criteria for sarcopenia in Asian individuals were determined, and sarcopenia was defined as having a L3-SMI<31.6 cm^2^/m^2^ in women and <40.2cm^2^/m^2^ in men (24).

### Statistical analysis

Descriptive statistics are reported as frequencies and percentages for categorical variables and medians and interquartile deviations (IQR) for continuous variables. Categorical variables were compared using chi-square tests. The relationship between baseline variables and the occurrence of irAEs was evaluated by logistic regression. Variables with P<0.2 in the unadjusted model were included in the multivariate analysis to explore independent risk factors associated with the occurrence of irAEs. To reduce the impact of baseline differences in demographic and clinical characteristics on outcomes, patients in the sarcopenia and non-sarcopenia groups were matched using a 1:1 propensity score matching (PSM) method. With the presence of sarcopenia as the dependent variable and each covariate as an independent variable, the basic principle of propensity scoring is to replace multiple covariates with a single score in order to equalize the distribution of covariates between the treatment and control groups. The ratio value was 1 while caliper value was 0.02. P<0.05 was considered to indicate a statistically significant difference. Statistical data analysis was carried out by SPSS software (version 27.0; IBM, Corp.).

## Results

The present study evaluated 129 patients with advanced lung cancer. **Table 1** summarizes the baseline characteristics of the participants. A total of 64.3% of participants were <65 years old, with a male/female patient ratio of 85/44. Pathological types included adenocarcinoma (62 patients), squamous cell carcinoma (36 patients) and neuroendocrine carcinoma (31 patients). The ECOG performance status (PS) was 0-1 in 70 patients (54.3%) and 2 in 59 patients (45.7%). According to the BMI classification, 33.3% of the participants were overweight or obese. 70 (54.3%) participants had PD-L1 expression ≥ 1%. Sarcopenia was identified in 79 (61.2%) participants based on the L3-SMI cut-off values for sarcopenia in Asian adults determined in a previous study (24).

**Table 1:** Baseline characteristics of total study population (N=129).

Characteristics	Sub-Groups	N	%
**Gender**	Female	44	34.1
	Male	85	68.9
**Age**	Median (Range)	60 (52-66)	
	<65	83	64.3
	≥65	46	35.7
**BMI**	Under weigh (BMI ≤ 18.5)	12	9.3
	Normal weight (18.5<BMI ≤ 24.9)	74	57.4
	Overweight and Obese (BMI≥25)	43	33.3
**ECOG PS**	0	24	18.6
	1	46	35.7
	2	59	45.7
**Histology**	Adenocarcinoma	62	48.1
	Squamous	36	27.9
	Neuroendocrine	31	24.0
**ICI type**	PD-1	95	73.6
	PD-L1	34	26.4
**Smoking**	Yes	63	48.8
	No	66	51.2
**Sarcopenia**	Yes	79	61.2
	No	50	38.8
Line of PD-1/PD-L1 therapy			
First		92	71.3
Second or above		37	28.7
Nutritional status			
Well nourished		47	36.4
Moderate or suspected malnutrition		55	42.6
Severe malnutrition		27	21.0
**PD-L1 expression**	<1%	59	45.7
	≥1%	70	54.3

**Figure 2:**
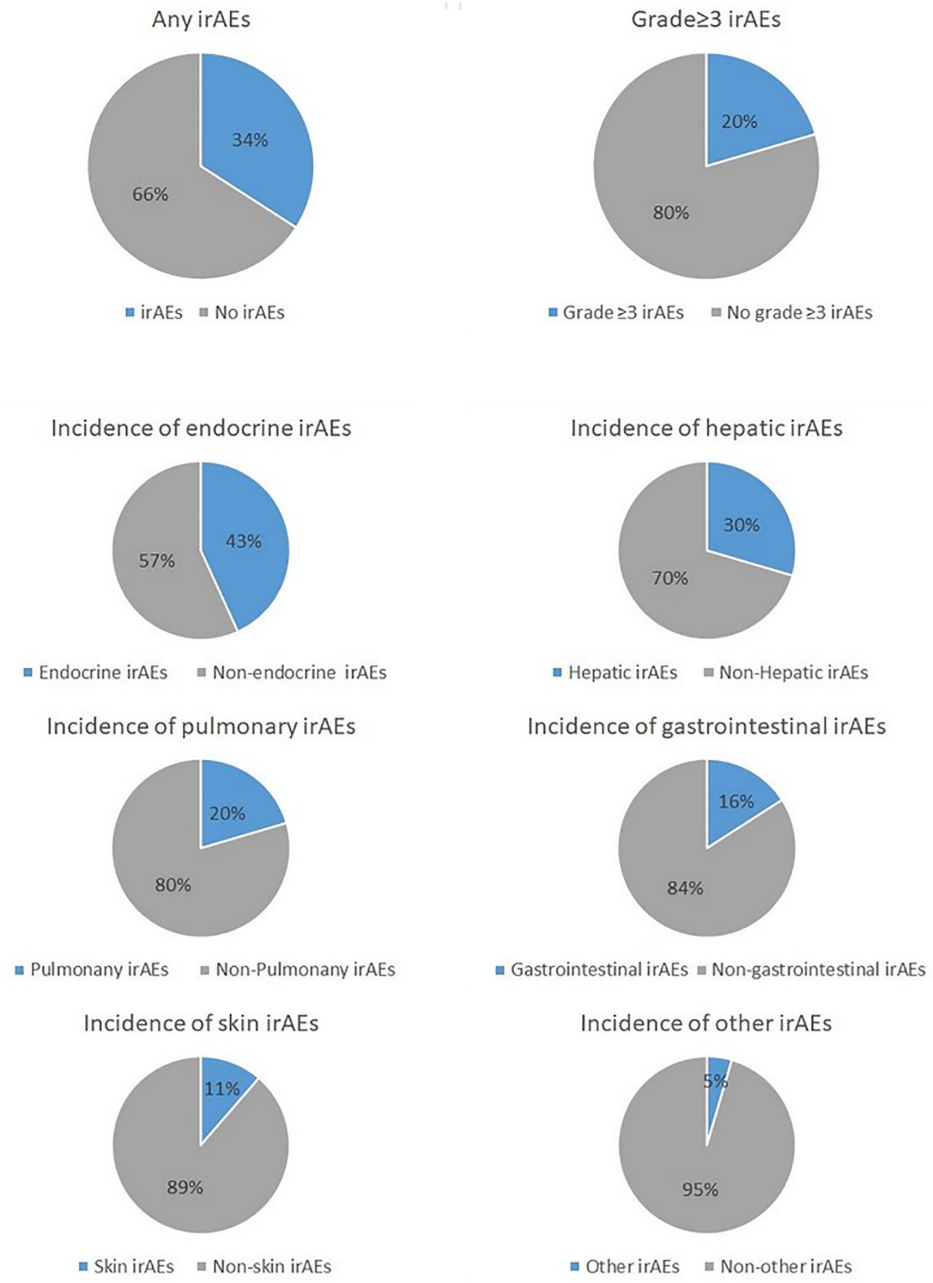
Incidence of irAEs and organ-specific irAEs.

Overall, 34% (n=44) of all participants presented with any grade of irAEs, which included 20% (n=9) who presented with grade 3 or higher irAEs. The most common irAE was endocrine-related (43%; n=19), followed by hepatic-related (30%; n=13), pulmonary-related (30%; n=9), gastrointestinal-related (16%; n=7), skin-related (11%; n=5) or other (5%; n=2). Of note, one single patient may have irAEs in different organ systems at the same time (**Figure 2**).

**Figure 3:**
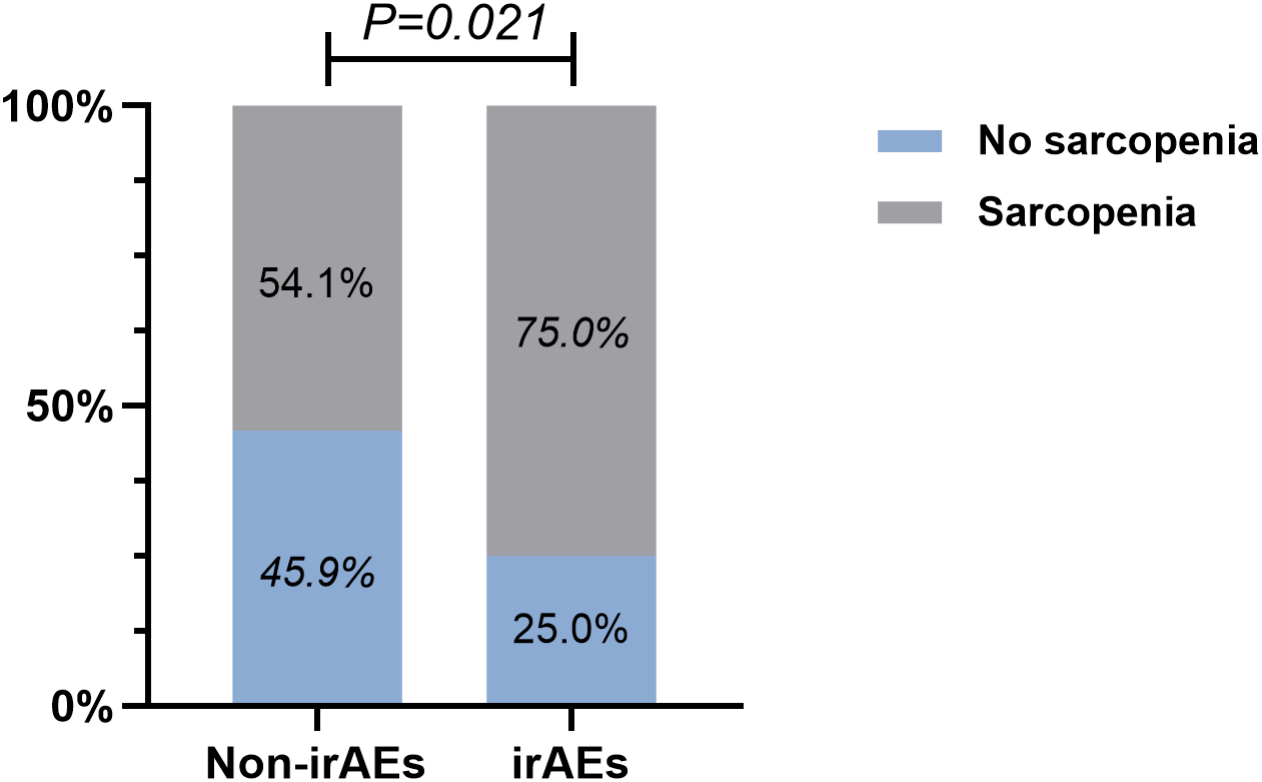
Relationship between sarcopenia and the occurrence of any grade irAEs.

**Figure 4:**
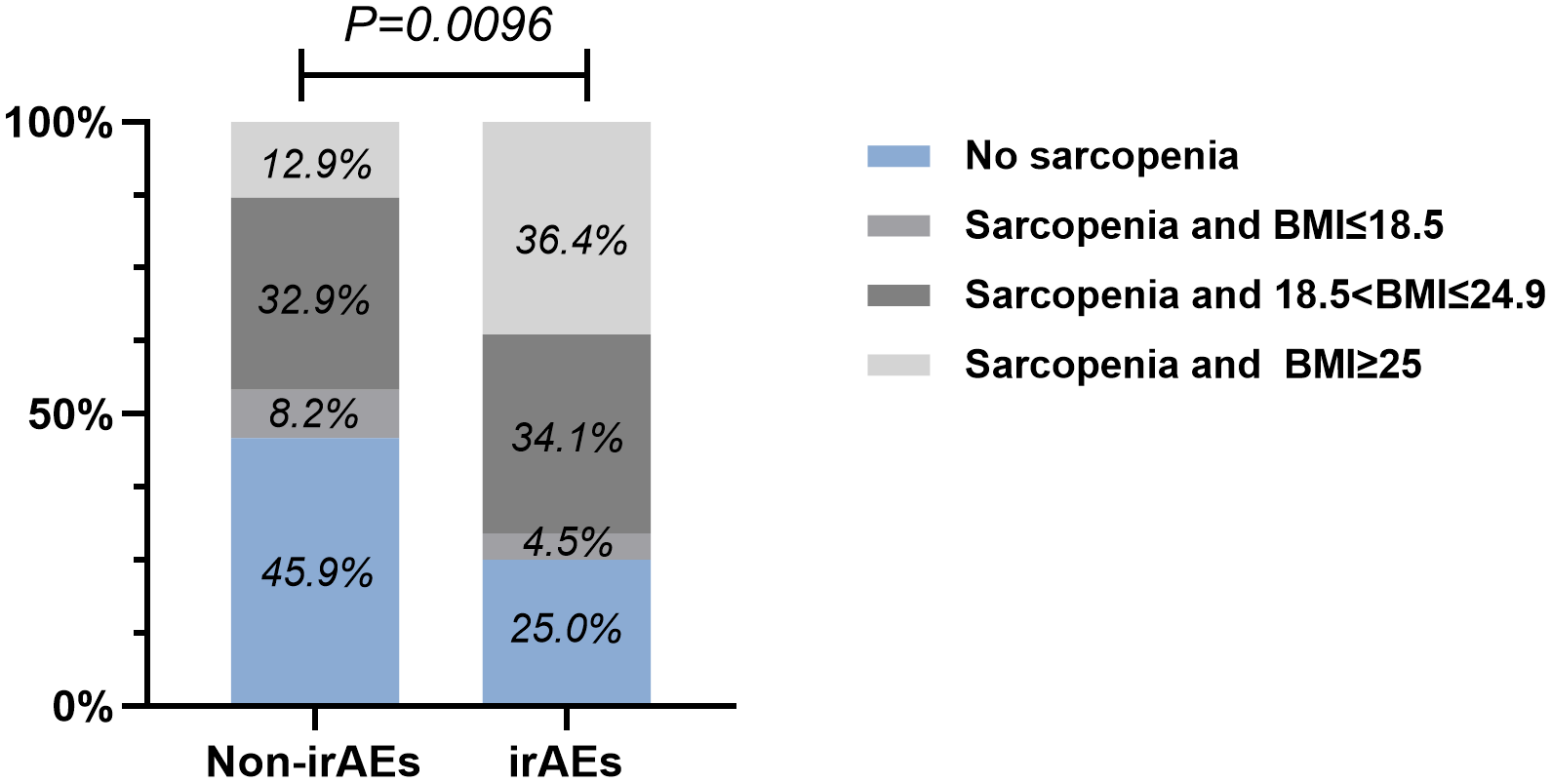
The relationship between sarcopenia, BMI, and the occurrence of any grade irAEs.

By performing chi-square analysis, the patient baseline characteristics that were associated with the occurrence of irAEs were assessed (**Table 2**). There was no significant difference between the irAEs and non-irAEs groups in terms of sex, age, ECOG-PS, histology type, Nutritional status and type of ICI used. Factors significantly associated with the occurrence of irAEs of any grade were BMI (P=0.032), PD-L1expression (P=0.022) and sarcopenia (P=0.021). The proportion of overweight and obese patients was significantly higher in the irAEs group than in the non-irAEs group. **Table 3** shows a comparison of baseline characteristics between sarcopenia and non-sarcopenia patients. There was a significant difference between the two groups in terms of nutritional status, which was worse in patients with sarcopenia. Also, we found that patients receiving later-line immunotherapy appeared to be more likely to develop sarcopenia, but this difference was not statistically significant. In addition, the present study revealed a relationship between sarcopenia and the occurrence of any grade irAEs. A total of 75.0% of patients in the irAEs group had combined sarcopenia, which was significantly higher than in the non-irAEs group (**Figure 3**). The proportion of patients with sarcopenia combined with overweight/obese was also significantly higher than in the non-irAEs group (**Figure 4**).

**Table 2:** Patient characteristics of the non-irAEs and irAEs groups.

Characteristics	Type of Participants		p-value
	Non-irAEs (n=85); N (%)	irAEs (n=44); N (%)	
**Age group**			0.611
<65	56 (65.9)	27 (61.4)	
≥65	29 (34.1)	17 (38.6)	
**Gender**			0.116
Female	33 (38.8)	11 (25.0)	
Male	52 (61.2)	33 (75.0)	
**BMI**			**0.032**
Underweight	10 (11.8)	2 (4.5)	
Normal weight	53 (62.4)	21 (47.7)	
Overweight and Obese	22 (25.9)	21 (47.7)	
**Performance status**			0.560
0	18 (21.2)	6 (13.6)	
1	30 (35.3)	16 (36.4)	
2	37 (43.5)	22 (50.0)	
**Histology**			0.393
Adenocarcinoma	39 (45.9)	23 (52.3)	
Squamous	27 (31.8)	9 (20.5)	
Neuroendocrine	19 (22.4)	12 (27.3)	
**ICI type**			0.544
PD-1	64 (75.3)	31 (70.5)	
PD-L1	21 (24.7)	13 (29.5)	
**Smoking**			0.192
Yes	38 (44.7)	25 (56.8)	
No	47 (55.3)	19 (43.2)	
**Line of PD-1/PD-L1 therapy**			0.571
First	62 (72.9)	30 (68.2)	
Second or above	23 (27.1)	14 (31.8)	
**Nutritional status**			0.630
Well nourished	33 (38.8)	14 (31.8)	
Moderate or suspected malnutrition	36 (42.4)	19 (43.2)	
Severe malnutrition	16 (18.8)	11 (25.0)	
**PD-L1 expression**			
<1%	45 (52.9)	14 (31.8)	**0.022**
≥ 1%	40 (47.1)	30 (68.2)	

Bold indicates statistically significant, P < 0.05.

**Table 3:** Patient characteristics of the no sarcopenia and sarcopenia groups.

Characteristics	Type of Participants		p-value
	No sarcopenia (n=50); N (%)	Sarcopenia (n=79); N (%)	
**Age group**			0.490
<65	34 (68.0)	49 (62.2)	
≥65	16 (32.0)	30 (38.0)	
**Gender**			0.261
Female	20 (40.0)	24 (30.4)	
Male	30 (60.0)	55 (69.6)	
**BMI**			0.520
Underweight	3 (6.0)	9 (11.4)	
Normal weight	31 (62.0)	43 (54.4)	
Overweight and Obese	16 (32.0)	27 (34.2)	
**Performance status**			0.676
0	8 (16.0)	16 (20.3)	
1	20 (40.0)	26 (32.9)	
2	22 (44.0)	37 (46.8)	
**Histology**			0.092
Adenocarcinoma	26 (52.0)	36 (45.6)	
Squamous	17 (34.0)	19 (24.1)	
Neuroendocrine	7 (14.0)	24 (30.4)	
**Line of PD-1/PD-L1 therapy**			0.182
First	39 (78.0)	53 (65.8)	
Second or above	11 (22.0)	26 (34.2)	
**Nutritional status**			**0.003**
Well nourished	27 (54.0)	20 (25.3)	
Moderate or suspected malnutrition	17 (34.0)	38 (48.1)	
Severe malnutrition	6 (12.0)	21 (26.6)	

Bold indicates statistically significant, P < 0.05.

To further explore the risk factors associated with the occurrence of irAEs, a multifactorial logistic regression analysis was performed (**Table 4**). After adjusting for confounding factors, sarcopenia was independently associated with the occurrence of irAEs. The odds ratio (OR) for the presence of irAEs in patients with sarcopenia was 2.635-times higher than in patients without sarcopenia [95% confidence interval (CI), 1.099-6.317; P=0.03]. It was also found that patients with high BMI (OR=2.737, 95% CI, 1.147-6.533; P=0.023) had an increased risk of irAEs than patients with a normal BMI. When PD-L1 expression is ≥ 1%, the risk of developing irAEs increases by 3.075(95% CI, 1.322-7.149; P=0.009) times. Similarly, the risk of irAEs in patients with sarcopenia combined with overweight/obesity was 5.502-fold higher after adjusting for confounders (Sex, Smoking, PD-L1 expression). To reduce the effect of bias we performed a 1:1 PSM analysis among the patient groups. The group without sarcopenia was used as the baseline group for matching, and ultimately, 88 patients were evaluated, with each subgroup comprising 44 patients. The P values for all covariates were greater than 0.05, indicating that propensity scores for the two groups significantly overlapped (**Table 5**). Multivariate analysis of propensity score-matched groups showed that sarcopenia ((OR=5.674, 95% CI, 1.506-21.380; P=0.01), PD-L1 expression ((OR=4.037, 95% CI, 1.0897-14.958; P=0.037) were significantly associated with the occurrence of irAEs (**Table 6**).

**Table 4:** Univariate and multivariate logistic regression analyses of factors associated with irAEs.

Clinical variables	Crude Odds 95% CI	Adjusted^a^ Odds 95% CI
Age		
<64	REF	——
≥ 65	1.216 (0.572-2.586)	——
Sex		
Female	REF	REF
Male	1.904 (0.847-4.279)	1.205 (0.40-3.156)
Smoking		
No	REF	REF
Yes	1.627 (0.781-3.390)	2.074 (0.821-5.241)
ICI type		
PD-1	REF	——
PD-L1	1.278 (0.566-2.884)	——
BMI		
Normal weight	REF	REF
Underweight	0.505 (0.102-2.500)	0.237 (0.042-1.347)
Overweight and Obese	**2.409 (1.101-5.271)**	**2.737 (1.147-6.533)**
Performance Status		
0	REF	——
1	1.600 (0530-4.832)	——
2	1.784 (0.616-5.169)	——
SMI		
No sarcopenia	REF	REF
Sarcopenia	**2.543 (1.137-5.688)**	**2.635 (1.099-6.317)**
Sarcopenia and BMI		
No sarcopenia	REF	REF
Sarcopenia and BMI ≤ 18.5	1.013 (0.184-5.590)	0.573 (0.092-3.554)
Sarcopenia and 18.5<BMI ≤ 24.9	1.899 (0.759-4.752)	1.882 (0.724-4.893)
Sarcopenia and BMI≥25	**5.157 (1.863-14.278)**	**5.502 (1.861-16.262)**
PD-L1 expression		
<1%	REF	REF
≥ 1%	**2.411 (1.123-5.176)**	**3.075 (1.322-7.149)**

CI, confidence interval; OR, odds ratio.

a Variables with p-values <0.2 in the univariate analysis were included in the multivariate analysis; The variables SMI and BMI were removed from the multivariate logistic analysis of Sarcopenia and BMI.

Bold indicates statistically significant, P < 0.05.

**Table 5:** Patient characteristics of the no sarcopenia and sarcopenia groups after propensity score matching.

Characteristics	Type of Participants		p-value
	No sarcopenia (n=44); N (%)	Sarcopenia (n=44); N (%)	
**Age group**			0.478
<65	30 (68.2)	33 (75.0)	
≥65	14 (31.8)	11 (25.0)	
**Gender**			0.381
Female	19 (43.2)	15 (34.1)	
Male	25 (56.8)	29 (65.9)	
**Smoking**			0.828
No	26 (59.1)	27 (61.4)	
Yes	18 (40.9)	17 (38.6)	
**ICI type**			0.777
PD-1	36 (81.8)	37 (84.1)	
PD-L1	8 (18. 2)	7 (15.9)	
**BMI**			0.520
Underweight	2 (4.5)	3 (6.8)	
Normal weight	27 (61.4)	26 (59.1)	
Overweight and Obese	15 (34.1)	15 (34.1)	
**Performance status**			0.694
0	6 (13.6)	9 (20.5)	
1	17 (38.6)	16 (36.4)	
2	21 (47.7)	18 (43.2)	
**Histology**			0.670
Adenocarcinoma	24 (54.5)	19 (43.2)	
Squamous	12 (27.3)	13 (29.5)	
Neuroendocrine	8 (18.2)	12 (27.3)	
**Line of PD-1/PD-L1 therapy**			0.758
First	36 (81.8)	35 (79.5)	
Second or above	8 (18.2)	9 (20.5)	
**PD-L1 expression**			
<1%	22 (50.0)	21 (47.7)	0.831
≥ 1%	22 (50.0)	23 (52.3)	
**Nutritional status**			0.533
Well nourished	15 (34.1)	15 (34.1)	
Moderate or suspected malnutrition	20 (45.5)	22 (50.0)	
Severe malnutrition	9 (20.5)	7 (15.9)	

**Table 6:** Univariate and multivariate logistic regression analyses of factors associated with irAEs after propensity score matching.

Clinical variables	Crude Odds 95% CI	Adjusted^a^ Odds 95% CI
Age		
<64	REF	REF
≥ 65	2.040 (0.688-6.045)	1.634 (0.444-6.007)
Sex		
Female	REF	REF
Male	2.586 (0.747-8.949)	2.361 (0.518-10.753)
Smoking		
No	REF	REF
Yes	2.486 (0.834-7.411)	1.493 (0.391-5.703)
ICI type		
PD-1	REF	——
PD-L1	1.120 (0.339-3.698)	——
BMI		
Normal weight	REF	——
Underweight	0.658 (0.233-3.752)	——
Overweight and Obese	1.790 (0.587-5.464)	——
Performance Status		
0 1 2	REF 0.921 (0.217-3.917) 1.000 (0.242-4.138)	—— —— ——
**SMI**		
No sarcopenia	REF	REF
Sarcopenia	**4.060 (1.261-13.072)**	**5.674 (1.506-21.380)**
Sarcopenia and BMI		
No sarcopenia	REF	REF
Sarcopenia and BMI ≤ 18.5	1.106 (0.149-6.423)	1.701 (0.145-4.524)
Sarcopenia and 18.5<BMI ≤ 24.9	3.867 (1.049-14.248)	5.025 (1.201-24.465)
Sarcopenia and BMI≥25	**6.960 (1.523-31.811)**	**8.843 (1.625-36.220)**
PD-L1 expression		
<1%	REF	REF
≥ 1%	2.368 (0.804-6.974)	**4.037 (1.089-14.958)**

Bold indicates statistically significant, P < 0.05.

## Discussion

In the present retrospective study, it was found that 34% (44/129) of participants had irAEs of any grade and 7.0% (9/129) had irAEs of ≥ grade 3. These findings are generally consistent with the findings of the aforementioned MATE analysis (9). There are various predictors of irAEs associated with anti-PD-1/PD-L1 therapy in clinical practice, such as BMI, autoimmune disease and neutrophil-to-lymphocyte ratio (11, 12, 14). However, a study involving patients with NSCLC treated with anti-PD-1 therapy has shown that sarcopenia is associated with the efficacy of anti-PD-1 therapy (17). Therefore, the present study investigated whether the presence of sarcopenia and other risk variables in patients with lung cancer increased the risk of irAEs in participants. Multiple baseline risk factors were included in this study.

We found that participants with sarcopenia at baseline had a 2-fold higher risk of irAEs on anti-PD-1/L1 therapy than participants without sarcopenia, and that the risk was 5-fold higher in patients with sarcopenia combined with overweight/obesity. Sarcopenia is a chronic progressive disease, and since the participants in the present study were primarily patients with advanced lung cancer, 61.2% developed sarcopenia, a higher incidence of sarcopenia than reported in prior research that included patients with cancer at any stage (25). High BMI has been shown to be strongly associated with the occurrence of adverse events of chemotherapy and immune-related treatments (12, 26), which is consistent with our findings. Currently, the mechanisms through which sarcopenia contributes to the occurrence of adverse events (AEs) remain largely unexplored. This condition has been associated with inflammation and insufficient nutritional status, which may help explain the heightened treatment-related toxicities and the generally poorer survival rates observed (27). Skeletal muscle cells express major histocompatibility complexes that activate T cells, so a reduction in muscle mass could disrupt the body’s homeostatic balance (27, 28). Additionally, the decline in myokines, particularly IL-15, can disturb the delicate equilibrium among various T-cell subsets, notably natural killer (NK) cells. This issue is exacerbated by the substitution of muscle tissue with adipose tissue, leading to the secretion of adipokines and a complicated network of signaling pathways that ultimately fosters an unregulated inflammatory response (29, 30). It is now also understood that patients with cancer do not necessarily lose or gain fat and skeletal muscle in equal proportions when their weight changes and indeed fat can be gained when muscle is being lost (31). Sarcopenia is a crucial factor in patients with cancer, according to a rising number of studies, whereby sarcopenia is strongly associated with the toxicity of standard chemotherapy and tyrosine kinase inhibitor (TKI) therapy (32, 33).

Evidence indicates that patients with cancer receiving ICI therapy have greater survival rates and improved treatment response rates when irAEs are present (34, 35). Also, the presence of sarcopenia was associated with poorer ICI treatment outcomes (17). Therefore, it is hypothesized that there is an association between sarcopenia and irAEs. This relationship is attributed to two main reasons. First, patients with sarcopenia treated with standard doses of monoclonal antibodies may be over-exposed, resulting in excessive toxicity. Of the three main treatment modalities currently available for patients with cancer, the dose of most cytotoxic drugs is related to body area (m^2^), the dose of TKIs is generally a flat dose and for most monoclonal antibodies, the dose is related to body weight. Sarcopenia is usually associated with increased fat mass (36). Clinically, the dose of monoclonal antibodies used increases with the amount of fat and does not adjust to changes in body composition, which ultimately leads to overdosing and an increase in adverse effects. This may also explain why sarcopenia can be considered an independent predictor of toxicity occurrence among patients treated for standard chemotherapy and TKIs (32, 33). Pharmacokinetic tests on patients with sarcopenia also support this hypothesis (37, 38). In view of the excessive toxicity tendency of patients with sarcopenia, some articles have pointed out that dose capping may be beneficial to these patients but there is no specific standard proportional adjustment scheme, and dose capping may impact the drug efficacy in obese patients with sarcopenia (39).

Second, this association may be the result of systemic inflammation. Inflammatory biomarkers have potential significance in predicting prognosis in cancer patients (40). Chronic systemic inflammation is the main cause of sarcopenia (36). Loss of muscle tissue disrupts the homeostasis of the body, and the secretion of adipokines in adipose tissue and complex muscle factor interactions lead to chronic systemic inflammation (41). Reportedly, patients with sarcopenia have abnormally increased T lymphocyte levels in their peripheral blood (42). The main mechanism by which irAEs occur is considered to be the activation of damage to the body’s own tissues due to the reaction of T lymphocytes with self-antigens after the administration of ICIs. Elevated lymph counts reflect an excessive anti-tumor response of the immune system and an increased risk of self-tissue damage. Consistent with the expected results, increases in lymphocyte counts during treatment were frequently observed in patients with melanoma (43). High lymphatic counts, and elevated interleukin-6 and C-reactive protein have been shown to be possible predictors of the development of irAEs (14, 44).

The association between sarcopenia and irAEs is complex, and reflecting this, the present study had conflicting results. Cortellini et al. (45) reported no statistically significant correlation between sarcopenia and irAEs, Furthermore, Haik et al. (46) also did not establish a link between the two. Inconsistent assessment methods for sarcopenia, among other reasons, may have contributed to this contradictory result. Due to the small number of studies, further research is needed to verify whether there is an association between sarcopenia and the risk of irAEs.

The present study has several limitations. First, the limited sample size of the present single-center retrospective analysis meant that a definitive conclusion could not be reached. Large-scale, multicenter, prospective and retrospective studies are needed in the future to demonstrate the association between sarcopenia and irAEs. Besides, the best time-point to collect CT images would be before starting treatment with ICIs, but there are a very small number of patients whose CT images are taken within one week of the start of treatment. Furthermore, there are various methods of assessing sarcopenia, and multiple assessment methods could be taken and compared in future studies to determine the most effective tool. Finally, as the present study excluded patients with active immune diseases and a history of immune diseases, as well as systemic use of steroids and immunosuppressants, it was not possible to determine whether autoimmune diseases would affect the occurrence of irAEs.

In conclusion, the findings of the present study suggest the potential use of sarcopenia in predicting the risk of irAEs in patients with advanced lung cancer. Screening for sarcopenia by CT, the most commonly used test, can help identify patients who may present with irAEs in clinical practice. However, improved methods and medications are required, such as protein supplements, resistance training or medicines like anamorelin, to increase muscle mass and function in patients with advanced NSCLC (47). Future studies should focus on other possible predictors of irAEs.

## Data Availability

The raw data supporting the conclusions of this article will be made available by the authors, without undue reservation.
